# Three-dimensional motion capture data during repetitive overarm throwing practice

**DOI:** 10.1038/sdata.2018.272

**Published:** 2018-12-04

**Authors:** Gizem Ozkaya, Hae Ryun Jung, In Sub Jeong, Min Ra Choi, Min Young Shin, Xue Lin, Woo Seong Heo, Mi Sun Kim, Eonho Kim, Ki-Kwang Lee

**Affiliations:** 1 Biomechanics and Sports Engineering Lab, Department of Physical Education, Kookmin University, Seoul, South Korea; 2 Korea Institute of Sport Science, Seoul, South Korea

**Keywords:** Motor control, Physiology, Learning and memory

## Abstract

Three-dimensional motion capture analysis is considered the gold standard for any movement research. Motion capture data were recorded for 7 healthy female participants with no prior throwing experience to investigate the learning process for overarm throwing during a selected period. Participants were monitored 3 times a week for 5 weeks. Each session consisted of 15 dominant and 15 nondominant hand side overarm throws. A total of 3,150 trials were recorded and preprocessed (labeling reflective markers) for further analysis. The presented dataset can provide valuable information about upper extremity kinematics of the learning process of overarm throwing without any kind of feedback. Furthermore, this dataset may be used for more advanced analysis techniques, which could lead to more insightful information.

## Background & Summary

Three-dimensional motion analysis systems, which use markers to track motion, are expensive, and they require long amounts of time. Therefore, due to the nature of the biomechanics research, most studies have had a highly limited number of trials, which makes it hard to use advanced data analysis methods, such as machine and deep learning. Most biomechanics studies with large numbers of data and researchers use educated guesses for data reduction before starting data acquisition, and they are limited to investigation of only several variables at specific events (e.g., shoulder flexion angle at ball release). With the advances of computers and computing methods, researchers have started to use more advanced analysis methods, such as principal component analysis, support vector machines, and regressions on larger datasets^1–8^. Studies that use more advanced analysis methods are mostly focused on cyclic movements such as gait and running. It has been shown that these methods may detect age-^2^ and fatigue-related^5^ changes, overall variability^3,4,7^, and the effects of different equipment^6^. However, limitations such as the sheer amount of rigorous work time and the financial support it takes to collect and process the data for the further calculations make it difficult for researchers to use more advanced analysis techniques.

Even though the use of reflective markers in 3D motion capture is very accurate (error < 1 mm), it is difficult to accurately track the shoulder and scapular joint movement. As the shoulder joint is connected to the scapular girdle, it has the ability to move in several planes simultaneously. This makes defining the shoulder joint biomechanics difficult and limited. Even with the work of the International Society of Biomechanics, a standard definition for shoulder biomechanics is not universally accepted^9^. Therefore, each research group uses its own model and marker set to define shoulder biomechanics according to the specific purpose of its study^10–12^. It was reported that rotation sequences have an effect on shoulder kinematics^13,14^. Some rotational sequences for the shoulder joint can result in gimbal lock occurrences more often than other combinations^13,14^. A modified marker set from other studies was used during data acquisition^10,11^. The aim of the modified marker set was for future studies to investigate differences of the upper extremity biomechanics’ definitions.

Another aspect of this study was to investigate the movement variability during the early skill acquisition for overarm throwing. In literature, extensive studies of upper extremity biomechanics during throwing activities^15–18^ and variability related to overarm throwing^19–21^ have been conducted. However, there has been no definitive conclusion about the role of variability, especially in sports biomechanics and performance^22^. Differences between the models and quantifications of variability are a source of discrepancy between results. It was reported that different variability quantifications showed different patterns, such as standard deviation increases while the coefficient in variation decreased^23^. In the traditional approach, less variability was concluded to be better because of the invariance of the performance, and the variability was regarded as the noise of the system^24^. However, recent studies showed that variability is not a noise of the system but can actually be useful^24,25^. Dynamical system theories applied to biomechanical datasets showed that variability is actually important for the human body as a system. It was reported that experts decrease their space-joint variability while increasing their trajectory variability^19^. Furthermore, the authors suggested that heavier balls can actually be used to achieve optimal variability for performance improvement of basketball free throws. Additionally, other studies showed that variability can be used to differentiate skill levels, injuries, and different populations^12,26,27^.

Therefore, healthy women participants with no prior overarm throwing experience or practice were recruited, and they performed several sessions for 5 weeks in a laboratory setting. They performed the throws with no feedback about their performance to make sure that they used only their sensory system for throwing the ball “as fast as possible”.

## Methods

### Participants

Eight female participants were recruited for this study. One participant was excluded because she injured herself during another activity during the experimental period, which left a total of seven participants for data acquisition. The study protocol was approved by the institutional review board of Kookmin University and was conducted according to the Helsinki declaration. The subjects gave their informed consent to participate in the study. All subjects were injury-free for both lower and upper extremities in the most recent six months, and they had no lower or upper extremity surgery in the last two years.

### Procedures

**Figure 1:**
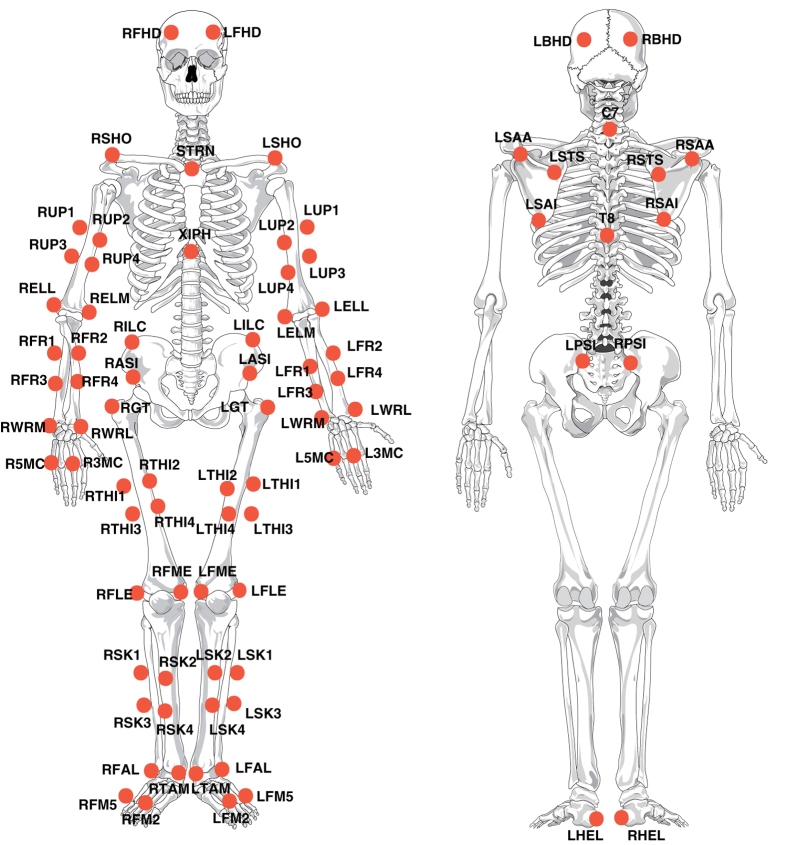
Experimental protocol.

Participants visited the laboratory three times a week for five weeks. All participants’ demographic and anthropometric measurements needed for modeling were carried out during the first session (Table 1). Each participant performed 15 dominant and 15 nondominant hand throws with a baseball. Participants were asked to throw the ball “as fast as possible” into a foam cushion (approx. 3 m x 3 m), which was approximately 4 m away from the participant. The first 3 throws for each side have been classified as a warm-up. Ball speeds were measured with a speed gun for each trial. The experimental procedure is shown in Fig. 1.

### Motion capture

Each session was performed in a laboratory fitted with 10 Vicon infrared cameras (T-10, T-40, Oxford Metrics Ltd., UK) and 2 force plates (BP-600900, AMTI, USA). Each camera connected to the Vicon MX Giganet and force plates were connected to the Giganet through a Vicon Datastation ADC patch panel. The giganet unit was connected to the main computer of the laboratory. Each of these cameras was equipped with an infrared strobe, which makes the reflective optical markers emit infrared light. Therefore, cameras recorded the trajectories of the markers via the emitted light from the markers. Marker trajectories were recorded through Vicon Nexus software (version 1.8.5, Oxford Metrics Ltd., UK). The marker trajectories and the force plate data were recorded synchronously at 200 and 1000 Hz sampling rates, respectively. A total of 450 trials were recorded for each participant.

**Figure 2:**
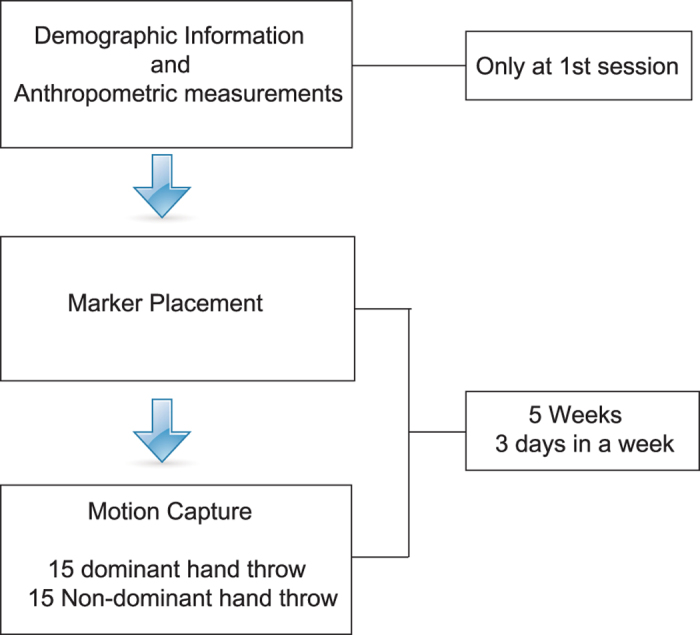
Marker set used in motion capture. Skeleton figures are taken from another source^29,30^.

At each session, participants wore tight clothes before the reflective marker placement. Participants performed warm-up throws until they became comfortable with the markers and ready to start. Markers and cluster markers (Fig. 2) affixed to the anatomical landmarks and extremities are listed in Table 2 (available online only). Static trials needed for modeling purposes in the further analysis were captured in two different positions. Dynamic trials were captured with only the tracking markers, as listed in Table 2 (available online only). All throws were made while participants had each foot placed on one force plate. The marker trajectories were saved automatically by the system without any names attached to them. For further calculations, each marker had to be labeled with a specific name. Each trials’ marker trajectories were labeled (the naming process of the markers according to Table 2 (available online only)) using Vicon Nexus software (version 1.8.5, Oxford Metrics Ltd., UK).

Each trial has three events: the start of the movement, the ball release, and the end of the movement. The start of the movement was defined as the moment when the distance between the trunk and the hand marker increased 10% more than the start position. The ball release was defined as the moment when the distance between the ball marker and the hand marker increased 10%. The end of the movement was selected as the moment of the end of the follow through and the closest distance between hand and trunk. Vicon Nexus software lets users label events with only three names: ‘Foot Strike’, ‘Foot Off’ and ‘General’. For future usage and to prevent confusion, the start of the movement event was labeled ‘Foot Strike’; the ball release, ‘General’; and the end of the movement, ‘Foot Off’.

## Data Records

c3d files are the standard file format that can be acquired from many motion capture systems and analyzed with different software. This file format is designed to include both three-dimensional point information and analog data. Therefore, each throw has its own c3d file that includes information about marker positions and all analog data. Additionally, comma-separated values (CSV) files, which include event times, positions of each marker, and force plate data, are included in the data records. The data records are available online from figshare (Data Citation 1), and they consist of c3d and CSV files and a detailed description file for each trial’s information (Trial Information, Data Citation 1).

Each subject has her own folder, and each subject folder has 15 folders which represent each session. Session folders were named ‘XwXd’, where X is the incremental integer for the week and day. For example, 1w1d represents the 1^st^ week-1^st^ day session.

Each session folder consists of c3d and CSV files for dominant and nondominant throw trials. Each trial was named systematically as ‘<S>_DP(or NDP)_<xx>_<m>’, where ‘S’ represents the subject and DP and NDP represent dominant and nondominant hand throws, respectively. ‘xx’ and ‘m’ are the incremental integer and information about each trial, respectively. For information about each trial, please refer to the Trial Information (Data Citation 1). Trial Information files (Data Citation 1) are provided due to the non-systematic incremental numbers of trials, ball speed, and other situations such as missing markers and errors. The key tab in a Trial Information file (Data Citation 1) consists of the meaning of each letter in ‘m’. For example, ‘S1_DP_01_O’ would represent Subject 1’s dominant hand first throw, and ‘O’ represents the trial having no problem.

## Technical Validation

Markers were mostly affixed to the participants’ bodies by the first author. In some sessions, markers were affixed by other researchers but always checked by the first author before the recordings. Camera calibration was performed before each session, and the settings (such as strobe intensity and the threshold for centroid fitting) were chosen for optimal marker visibility and noise reduction.

Gap filling is a process used when there is a missing marker during the trial. It is used from the start to the end event for each trial. Gap filling via spline or pattern fill in Vicon Nexus software was used depending on which one concludes with the appropriate trajectory for movement. Experienced researchers execute gap filling during the labeling process. Some trials had missing markers from the start of the recording. Therefore, gap filling could not be performed in these trials. All trials were checked after labeling and the gap filling process by the first author. The Trial Information (Data Citation 1) includes information about each trial, whether or not it had any issues during acquisition or processing. The related description about the Trial Information (Data Citation 1) has been given in the Data Records.

## Usage Notes

For more detailed information about c3d files and their possible uses with different software, refer to the c3d website (https://www.c3d.org). The modified marker set can be described as the combination of two previously published studies^10,11^. An upper extremity kinematics study based on this dataset has been published^28^. Furthermore, the needed anthropometric measurements for this modeling can be found in Table 1. There are two static trials for each session in case researchers want to use different definitions of upper extremity biomechanics. Additionally, researchers should be mindful of the data because the gap filling was made from the events of the start and end of the movement. Therefore, gap filling was not performed in frames other than those of the duration of the throwing. Researchers should be mindful of the trials with missing markers because they may affect the results, depending on the chosen model for further analysis.

## Additional information

**How to cite this article**: Ozkaya, G. *et al*. Three-dimensional motion capture data during repetitive overarm throwing practice. *Sci. Data*. 5:180272 doi: 10.1038/sdata.2018.272 (2018).

**Publisher’s note**: Springer Nature remains neutral with regard to jurisdictional claims in published maps and institutional affiliations.

## Supplementary Material



## Figures and Tables

**Table 1:** Demographic information of subjects.

Subject	Age (Years)	Weight (kg)	Height (cm)	Wrist Width (mm)	Elbow Width (mm)	Knee Width (mm)	Ankle Width (mm)	Leg Length (cm)	Right Shoulder Offset (mm)	Left Shoulder Offset (mm)	Hand Thickness (mm)	Right Shoulder Circumference (cm)	Left Shoulder Circumference (cm)	Dominant Hand
S1	23	54.6	165	44	55	86	62	85	80	84	22	36.5	36.5	Right
S2	26	54.8	160	43	50	85	54	82.5	83	83	16	35.3	35.3	Left
S3	24	56.6	162	46	54	88	59	83.5	86	87	13	36.5	36.5	Right
S4	30	57.1	159	47	53	87	59	82.5	73	73	18	37.5	37.5	Right
S5	26	73.1	165	42	55	97	55	87.3	92	95	17	40.5	40.5	Right
S6	23	51.1	160	40	50	85	55	82.2	61	63	27	34.3	34.3	Right
S7	24	48.7	155	45	50	87	56	80.6	81	81	14	35.3	35.3	Right
Mean±SD	25.1±2.4	56.5±7.8	160.8±3.5	43.8±2.4	52.4±2.3	87.8±4.1	57.1±2.9	83.3±2.1	79.4±9.9	80.8±10.2	18.1±4.8	36.5±2.0	36.5±2.0	

**Table 2:** Marker set used in motion capture.

Marker Name	Segment/Joint	Location	Tracking
LFHD	Head	Apprx. over the Left Temple	X
RFHD	Head	Apprx. over the Right Temple	X
LBHD	Head	Left Back of the Head	X
RBHD	Head	Right Back Of The Head	X
STRN	Trunk	Suprasternal Notch	X
XIPH	Trunk	Xiphoid Process	X
C7	Trunk	7^th^CervicalVertebra	X
T8	Trunk	8^th^ThoracicVertebra	X
RASI	Pelvis	Right Ant Superior Iliac Crest	X
RPSI	Pelvis	Right Post Superior Iliac Crest	X
RILC	Pelvis	Right Iliac Crest	X
LASI	Pelvis	Left Ant Superior Iliac Crest	X
LPSI	Pelvis	Left Post Superior Iliac Crest	X
LILC	Pelvis	Left Iliac Crest	X
RSHO	Right Humerus	Acromion Process	X
RSTS	Right Humerus	Trigonum Scapulae	X
RSAI	Right Humerus	Angulus Inferior	X
RSAA	Right Humerus	Angulus Acromialis	X
RUP1	Right Humerus	Upper Arm Cluster	X
RUP2	Right Humerus	Upper Arm Cluster	X
RUP3	Right Humerus	Upper Arm Cluster	X
RUP4	Right Humerus	Upper Arm Cluster	X
RELL	Right Elbow Joint	Lateral Epicondyle	
RELM	Right Elbow Joint	Medial Epicondyle	
RFR1	Right Forearm	Forearm Cluster	X
RFR2	Right Forearm	Forearm Cluster	X
RFR3	Right Forearm	Forearm Cluster	X
RFR4	Right Forearm	Forearm Cluster	X
RWRL	Right Wrist	Lateral Point of Radial Styloid	
RWRM	Right Wrist	Medial Point of Ulnar Styloid	
R3MC	Right Hand	3^rd^MetacarpalHead	X
R5MC	Right Hand	5^th^MetacarpalHead1	X
LSHO	Left Humerus	Acromion Process	X
LSTS	Left Humerus	Trigonum Scapulae	X
LSAI	Left Humerus	Angulus Inferior	X
LSAA	Left Humerus	Angulus Acromialis	X
LUP1	Left Humerus	Upper Arm Cluster	X
LUP2	Left Humerus	Upper Arm Cluster	X
LUP3	Left Humerus	Upper Arm Cluster	X
LUP4	Left Humerus	Upper Arm Cluster	X
LELL	Left Elbow Joint	Lateral Epicondyle	
LELM	Left Elbow Joint	Medial Epicondyle	
LFR1	Left Forearm	Forearm Cluster	X
LFR2	Left Forearm	Forearm Cluster	X
LFR3	Left Forearm	Forearm Cluster	X
LFR4	Left Forearm	Forearm Cluster	X
LWRL	Left Wrist	Lateral Point Of Radial Styloid	
LWRM	Left Wrist	Medial Point Of Ulnar Styloid	
L3MC	Left Hand	3^rd^MetacarpalHead	X
L5MC	Left Hand	5^th^MetacarpalHead1	X
RGT	Right Femur	Femur Greater Trochanter	X
RTH1	Right Femur	Thigh Cluster	X
RTH2	Right Femur	Thigh Cluster	X
RTH3	Right Femur	Thigh Cluster	X
RTH4	Right Femur	Thigh Cluster	X
RFLE	Right Knee Joint	Femur Lateral Epicondyle	
RFME	Right Knee Joint	Femur Medial Epicondyle	
RSK1	Right Shank	Shank Cluster	X
RSK2	Right Shank	Shank Cluster	X
RSK3	Right Shank	Shank Cluster	X
RSK4	Right Shank	Shank Cluster	X
RFAL	Right Ankle Joint	Fibula Lateral Malleolus	
RTAM	Right Ankle Joint	Tibia Medial Malleolus	
RFM2	Right Foot	2^nd^MetatarsalHead	X
RFM5	Right Foot	5^th^MetatarsalHead	X
RHEL	Right Foot	Posterior of Calcaneus	X
LGT	Left Femur	Femur Greater Trochanter	
LTH1	Left Femur	Thigh Cluster	X
LTH2	Left Femur	Thigh Cluster	X
LTH3	Left Femur	Thigh Cluster	X
LTH4	Left Femur	Thigh Cluster	X
LFLE	Left Knee Joint	Femur Lateral Epicondyle	
LFME	Left Knee Joint	Femur Medial Epicondyle	
LSK1	Left Shank	Shank Cluster	X
LSK2	Left Shank	Shank Cluster	X
LSK3	Left Shank	Shank Cluster	X
LSK4	Left Shank	Shank Cluster	X
LFAL	Left Ankle Joint	Fibula Lateral Malleolus	
LTAM	Left Ankle Joint	Tibia Medial Malleolus	
LFM2	Left Foot	2^nd^MetatarsalHead	X
LFM5	Left Foot	^5th^MetatarsalHead	X
LHEL	Right Foot	Posterior of Calcaneus	X
Ball	Baseball		X
